# Editing the 19 kDa alpha‐zein gene family generates non‐*opaque2*‐based quality protein maize

**DOI:** 10.1111/pbi.14237

**Published:** 2023-11-21

**Authors:** J. Preston Hurst, Shirley Sato, Tyler Ferris, Abou Yobi, You Zhou, Ruthie Angelovici, Tom E. Clemente, David R. Holding

**Affiliations:** ^1^ Department of Agronomy and Horticulture University of Nebraska‐Lincoln Lincoln Nebraska USA; ^2^ Center for Plant Science Innovation Lincoln Nebraska USA; ^3^ University of Missouri‐Columbia Columbia Missouri USA

**Keywords:** opaque2, CRISPR/cas9, seed storage proteins, QPM, zeins, lysine

## Abstract

Maize grain is deficient in lysine. While the *opaque2* mutation increases grain lysine, *o2* is a transcription factor that regulates a wide network of genes beyond zeins, which leads to pleiotropic and often negative effects. Additionally, the drastic reduction in 19 kDa and 22 kDa alpha‐zeins causes a floury kernel, unsuitable for agricultural use. Quality protein maize (QPM) overcame the undesirable kernel texture through the introgression of modifying alleles. However, QPM still lacks a functional *o2* transcription factor, which has a penalty on non‐lysine amino acids due to the *o2* mutation. CRISPR/cas9 gives researchers the ability to directly target genes of interest. In this paper, gene editing was used to specifically target the 19 kDa alpha zein gene family. This allows for proteome rebalancing to occur without an *o2* mutation and without a total alpha‐zein knockout. The results showed that editing some, but not all, of the 19 kDa zeins resulted in up to 30% more lysine. An edited line displayed an increase of 30% over the wild type. While not quite the 55% lysine increase displayed by QPM, the line had little collateral impact on other amino acid levels compared to QPM. Additionally, the edited line containing a partially reduced 19 kDa showed an advantage in kernel texture that had a complete 19 kDa knockout. These results serve as proof of concept that editing the 19 kDa alpha‐zein family alone can enhance lysine while retaining vitreous endosperm and a functional *O2* transcription factor.

## Introduction

Despite its status as the most produced crop on the planet, maize grain is deficient in lysine and tryptophan (Sauberlich *et al*., 1953). The poor protein quality is caused by the primary seed storage protein, the zeins, lacking lysine residues (Yau *et al*., 1999). Mutants of *opaque2* (*o2*) have a reduction in zein proteins and a corresponding increase in lysine‐rich non‐zein proteins through a phenomenon known as proteome rebalancing (Larkins *et al*., 2017). A soft kernelled, opaque maize mutant was first described by Jones and Singleton in the 1920's (Prasanna *et al*., 2001). In 1964, Mertz et al reported that this mutant, *o2*, contained twice the amount of lysine as WT maize (Mertz *et al*., 1964). However, the chalky kernel which caused this mutant to be noticed presented challenges to practical adoption. Driven by the promise of alleviating quality protein deficiencies in countries where animal protein can be more limited, researchers at CIMMYT used a recurrent selection scheme to breed maize lines that contain *o2* (Prasanna *et al*., 2001). These lines were classified as quality protein maize (QPM), and their development was the subject of the 2000 World Food Prize awarded to Dr. Surinder Vasal and Dr. Evangelina Villegas. QPM lines have had *o2* introgressed, along with kernel modifying alleles at other loci, such that they possess the high lysine trait characteristic of *o2* yet retain a hard kernel, due to the effects of the modifying alleles. However, QPM lines have been observed to typically have reduced yield and increased disease susceptibility compared to normal maize (Tandzi *et al*., 2017). Reduced yield is partially due to O2's role in regulating starch biosynthesis (Zhang *et al*., 2016). In addition, most of the modifying loci are unknown, making the breeding process laborious and lengthy. As the nature of the *o2* mutation was studied further, it became clear that *o2* encodes a bZIP transcription factor which regulates a diverse array of processes beyond just seed storage proteins, including genes related to pathogen response and nitrogen metabolism along with other transcription factors who themselves regulate other processes (Li *et al*., 2015; Schmidt *et al*., 1990; Zhan *et al*., 2018). O2 regulates the transcription of alpha‐zein genes, along with prolamine‐box‐binding‐factors (PBF1) and ZmMADS47 (Qiao *et al*., 2016; Vicente‐Carbajosa *et al*., 1997; Wang *et al*., 1998; Zhang *et al*., 2015).

Additionally, *o2* is involved in the transcription of gamma‐zeins and beta‐zeins (Neto *et al*., 1995). As zeins are the primary storage protein in maize kernels, the reduction seen in *o2* maize significantly alters the proteomic makeup. Importantly, however, the total amount of protein is unchanged. Instead, *o2* kernels accumulate different proteins. These other proteins contain higher lysine and tryptophan levels, providing the basis for the quality protein phenotype. Proteome rebalancing has been observed in other species. In soybean seeds, RNAi suppression of glycinin and conglycinin, the primary soybean storage proteins, results in an altered proteome without reduction in total protein (Schmidt *et al*., 2011). RNAi suppression of globulin storage protein in camelina also exhibited proteome rebalancing, and resulted in an increase in total protein (Schmidt and Pendarvis, 2017), while editing the crucifirin gene family did not change the total protein content yet did induce changes in proteome composition (Lyzenga *et al*., 2019). The *o2* transcription factor also regulates a range of other processes (Li *et al*., 2015; Zhan *et al*., 2018). RNAseq analysis of developing kernels displayed 1863 genes differentially expressed between B73 and B73*o2*, including 186 that were verified as direct targets via ChIPseq (Zhan *et al*., 2018). Many direct targets of *o2* are other transcription factors, themselves regulating other processes and creating a wide network of genes indirectly regulated by *o2* and thus collateral damage in *o2* mutant kernels. ]id=JPH]For example, *ZmGRAS11* is an O2 regulated transcription factor involved with cell expansion (Ji *et al*., 2022; Li *et al*., 2021). While the pleiotropic effects of *o2* make it unfavourable as a trait for agronomic reasons, it's important to recognize that a mutant in *o2* can reduce the expression of the entire alpha‐zein gene family. Given that there are >40 gene copies, it would have been highly unlikely to find a naturally occurring or even chemically induced mutant of multiple alpha‐zein genes. Thus, the reason *o2* has been highly important to the development of quality protein maize is that it is a single locus that regulates a large family of genes.

Zeins, the primary storage protein in maize grain, is made up of multiple subfamilies. The alpha‐zeins are the most abundant, making up 70% of the total zein protein fraction, and are divided into two subclasses based on SDS‐PAGE mobility: the 19 kDa and the 22 kDa. Further, these can be broken down into four subfamilies as a result of tandem duplication (Dong *et al*., 2016): The z1A, z1B, and z1D within the 19 kDa subclass, while the z1C family makes up the 22 kDa subclass. The 22 kDa, z1C family is on chromosome 4 and consists of 16–20 gene duplicates, depending on the specific inbred line being observed. It is linked to the 19 kDa z1A family, also chromosome 4. The z1A family contains 12 gene copies, across two linked loci. The z1B family, located on chromosome 7, is made up of eight copies. Additionally, the z1B locus has some linkage to the 27 kDa gamma‐zein locus, a locus important to QPM. The oldest alpha‐zein locus, the z1D family on chromosome 1, consists of five gene copies which are believed to be pseudogenes.

Zein proteins are ultimately incorporated into ER‐localized protein bodies. The mRNAs are directed to the ER via an N‐terminal signal peptide (Gillikin *et al*., 1997). As the protein body matures, the abundant alpha‐zeins fill the protein body's inner core. Ultimately, a mature protein body consists of an outermost layer of gamma‐zeins, and a two‐layer core consisting of the 22 and 19 kDa alpha zeins in distinct layers. The most inner core layer is made up of 19 kDa alpha‐zeins, surrounded by a layer of the 22 kDa subclass. RNAi constructs which target the 22 kDa alpha‐zein contain protein bodies which display lobing, or protrusion, disrupting the normally spherical shape (Segal *et al*., 2003; Wu and Messing, 2010). However, RNAi targeting both the 22 kDa and the 19 kDa simultaneously have normally formed, spherical protein bodies which are reduced in size (Guo *et al*., 2013). Presumably, the 22 kDa alpha‐zein is integral to the structural formation of protein bodies and has a stronger interaction with the gamma and beta zeins, while both the 19 kDa and the 22 kDa contribute to the size of the protein body via their role as filling the core (Gibbon and Larkins, 2005). The 27 kDa gamma‐zein has an important role in protein body formation as well (Liu *et al*., 2016, 2019; Wu *et al*., 2010). RNAi constructs targeting the 27 kDa gamma‐zein contain sparse amounts of protein bodies, with major defects in their shape especially when combined with beta‐zein RNAi (Guo *et al*., 2013; Wu and Messing, 2010). In kernels with suppressed 27 kDa gamma zeins, the protein bodies display a loss of structural integrity as seen by extensive lobing and loss of spherical conformation. Additionally, the sparseness, or reduced number of protein bodies is also characteristic of 27 kDa gamma zein suppression. Conversely, an increased dosage of the 27 kDa gamma zein via duplication corresponds to an increase in the number of protein bodies, owing to its role of initiating the formation of protein bodies (Liu *et al*., 2016, 2019). In fact, a duplication of the 27 kDa is the most well understood of the *o2* modifying loci introgressed into QPM. While the protein bodies are smaller because of a lack of alpha‐zeins, increased gene dosage of 27 kDa gamma‐zein increases the number of protein bodies formed. This contributes to the recovery of kernel hardness in maize lines carrying the *o2* mutation, resulting in QPM.

QPM was developed by using a recurrent selection scheme which introgressed *o2* modifying loci (Mo2s) from tropical composite lines into temperate maize backgrounds, while also maintaining a homozygous *o2* allele (Li *et al*., 2020). While the Mo2s were largely unknown during initial breeding, post hoc research has explored potential candidates. Primarily, a duplication of the aforementioned 27 kDa gamma zein has been validated as an Mo2 allele (Liu *et al*., 2016; Wu *et al*., 2010). Genetic analysis of segregating populations has proposed other candidate Mo2s related to ethylene and ABA biosynthesis as well as a pyrophosphate‐dependent fructose‐6‐ phosphate 1‐phosphotransferase (PFP) (Holding *et al*., 2008, 2011). Further analysis demonstrated an increase in PFP‐alpha subunit protein levels in QPM kernels relative to non‐QPM *o2* maize (Guo *et al*., 2012). Recently, a reference genome of QPM line K0326Y was assembled and associated analysis discovered a helitron insertion upstream PFPa, and a lack of multiple large retrotransposons which are present in B73 and Mo17 (non‐QPM) inbred lines. These structural variants are potentially responsible for the increased transcription observed in modified lines (Li *et al*., 2020). It has been hypothesized that increased PFPa contributes to QPM by making up for some of the ATP lost due to the *o2* mutations affects on metabolism via increased glycolytic flux (Guo *et al*., 2012; Li *et al*., 2020). Other Mo2 candidates include heat shock proteins and genes related to unfolded protein response, spread across the genome. QPM protein has a biological value of 90% that of milk, a great benefit to maize‐reliant people in parts of the world where animal protein was historically more scarce (Bressani *et al*., 1990; Prasanna *et al*., 2001). Today, as balanced diets around the world have improved, high‐lysine maize varieties hold particular value in livestock production. Monogastric livestock, such as swine, require lysine supplementation if raised on cereal‐based feed. Highly sine maize has been shown to replace the need for supplementation. For example, QPM fed to swine was shown to decrease the required soymeal relative to normal maize (Burgoon *et al*., 1992). When compared directly to a pure normal maize diet, QPM results in a nearly 10% increase in swine weight gain (Kemm *et al*., 1977). While QPM is grown in many countries and has had a positive impact, the loss of function mutation in *o2* still conveys negative pleiotropy. While kernel hardness is recovered, O2's broad regulatory network impacts disease susceptibility as well as yield (Prasanna *et al*., 2001; Tandzi *et al*., 2017).

Directly reducing transcription of the alpha‐zein gene provides an outlet for developing QPM containing a functional *o2* transcription factor. Several prior studies have utilized RNAi to target alpha‐zeins (Huang *et al*., 2004, 2006; Segal *et al*., 2003; Wu and Messing, 2010). RNAi targeting the total alpha‐zein family, 19 and 22 kDa subclasses, was bred into lines from the Illinois High Protein experiment and displayed enhanced lysine without losing a hard endosperm (Wu and Messing, 2012). However, RNAi requires a transgenic construct to be present in order to confer desired phenotype. This element of RNAi creates regulatory and economic hurdles, particularly in countries where QPM would be the most beneficial. Gene editing provides a pathway to developing high‐lysine maize varieties, which are not only transgene‐free but also contain a functional *o2* transcription factor. As previously noted, the 19 kDa alpha‐zeins make up the inner core of the protein, surrounded by the 22 kDa zeins. RNAi specifically targeting the 22 kDa class results in abnormally shaped protein bodies (Segal *et al*., 2003). Huang et al targeted the 19 kDa using a specific RNAi and did observe increases in lysine despite the 22 kDa alpha zein being unaffected and possibly increased via rebalancing (Huang *et al*., 2004). In the present study, CRISPR/cas9 was used to edit the 19 kDa subclass of the alpha‐zein gene family. By targeting the 19 kDa subclass only, the goal is to achieve proteome rebalancing while causing minimal structural degradation to the protein body. Leveraging six gRNA's, multiple target sites were selected across the z1A (Chr04) and z1B (Chr07) subfamilies, a total of 21 individual gene copies. Prior analysis determined the z1D(Chr01) subfamily was not targeted. Transcript abundance, gene body location, and mismatch location were utilized to identify the optimal set of six gRNAs capable of editing several gene copies. Primary transformants were outcrossed to a maize line containing a duplication of the 27 kDa gamma‐zein, a known Mo2 allele, to account for potential loss of vitreousness. The edited alleles could be selected for via PCR, and ultimately transgene‐free lines were produced. Additionally, the segregation of z1A and z1B loci allowed more specific knockout lines to be developed, a feature not available with RNAi. These lines have been demonstrated to have increased lysine, while also maintaining a vitreous endosperm. The result is a demonstration that transgene‐free, non‐*opaque2‐*based QPM can be developed by direct editing of an alpha‐zein subfamily.

## Results

### Target site selection and homozygous allele development

**Figure 1:**
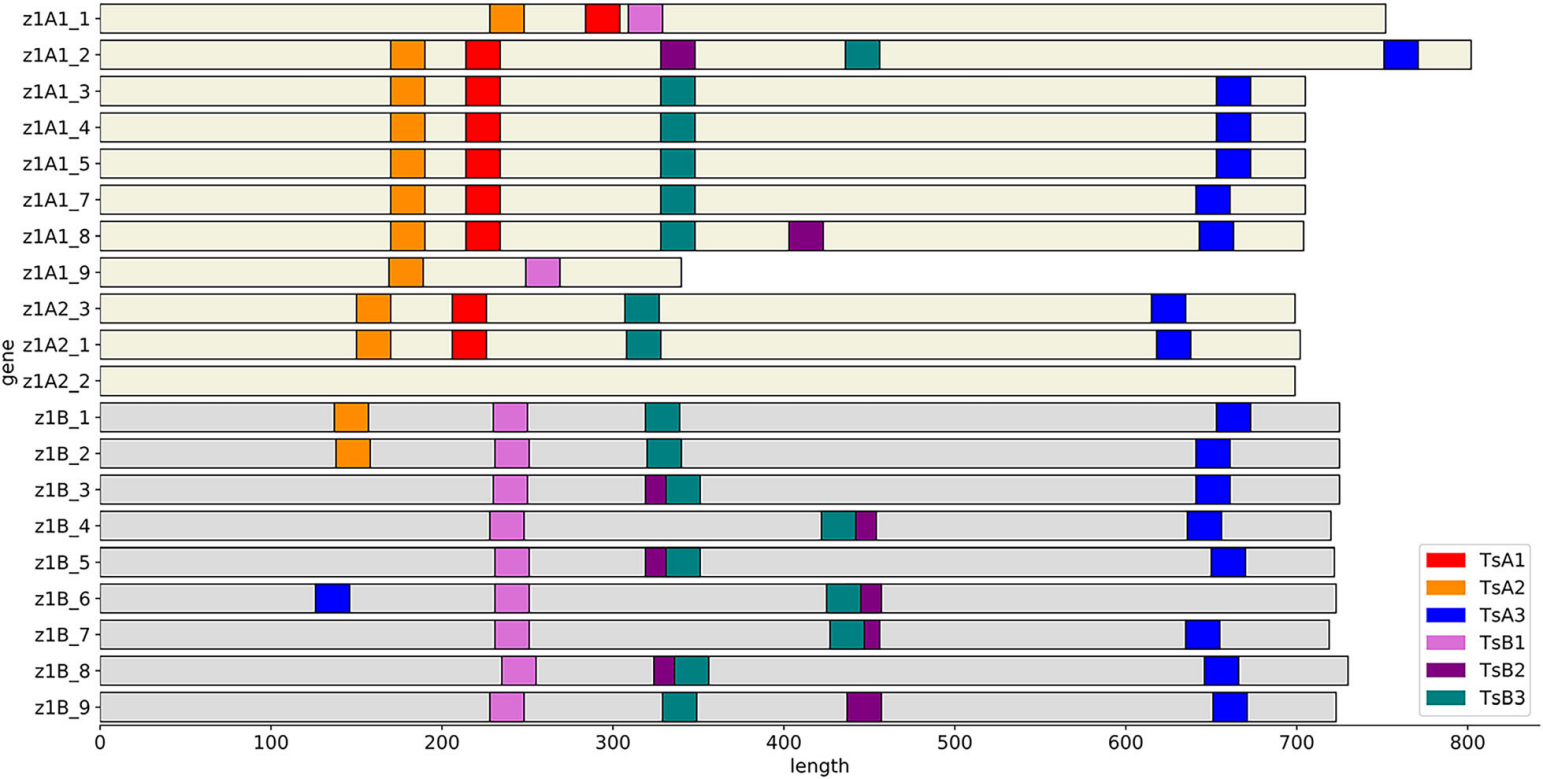
Six gRNA sequences were used to edit multiple sites in the 19 kDa gene bodies. Target sites were selected that could effectively edit multiple locations, such that large deletions would be made that facilitate the use of PCR primers for genotyping and breeding. Sites noted here indicate a seed sequence match, or the 12 bp adjacent to the PAM.

**Figure 2:**
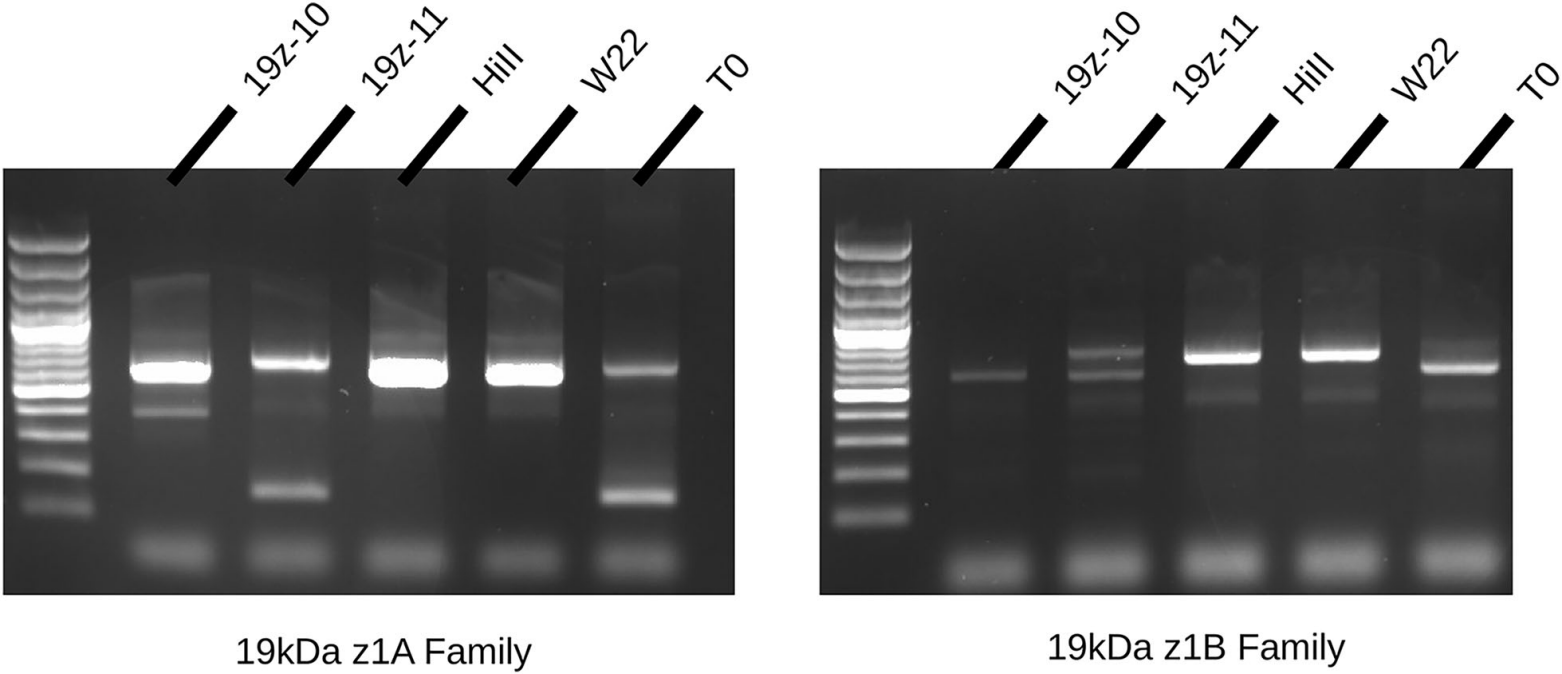
PCR markers were used for identifying edited alleles. Separate markers were used to identify edits in each gene family. Both reactions are amplifying multiple individual gene copies. HiII and W22 wild‐type inbreds display a single band, whereas 19z‐10 and 19z‐11 display smaller and multiple bands due to family members being edited.

As the alpha‐zein gene family is made up of many gene copies partitioned into separate subfamilies, it was important to identify which copies are expressed and to what degree. Previously, an RNAseq study compared zein transcription between two inbred lines with differing alpha‐zein haplotypes, B73 and W22 (Hurst *et al*., 2021). The results were used to prioritize the specific gene copies which dominate the expression profile, in order to ensure complete knock‐out and avoid residual expression compromising the hypothesized phenotype. Of note, the oldest family of 19 kDa gene copies, the D family on chromosome 1, were determined to be pseudogenes and not considered viable target genes (Dong *et al*., 2016; Hurst *et al*., 2021). All putative target sites, defined as a 20 bp string with a 5′‐NGG‐3′ adjacent motif, were compared in a pair‐wise fashion. Putative gRNAs were narrowed down to those that had a perfect 20 bp match to high‐priority gene copies. Additional filtering removed those which have a high off‐target potential. Using the reduced list, a set of six gRNA sequences (Table S1) was selected such that all high‐priority genes could be edited between two to five locations throughout the gene body (Figure 1). PCR primers capable of amplifying multiple gene copies were designed and used to identify individuals carrying the edited allele. Two markers were used, one for the z1A family members, and another for the z1B members. wild type alleles produce a single band, whereas an edited allele produced smaller or multiple bands due to the deletion of base pairs in between gRNA sites (Figure 2).

**Figure 3:**
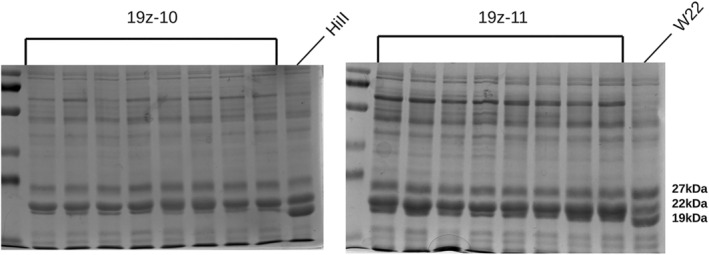
F2:F3 kernels demonstrated homozygosity and effective knockout of the 19 kDa alpha zein. SDS‐PAGE of individual kernels was used to identify which F2 plants were homozygous. Additionally, it was critical that the 19 kDa alpha zeins were affected.

T0 primary transformants, which were determined to contain an edited allele in both z1A and z1B, were crossed to inbred line W22 in order to increase the probability of recovering a vitreous endosperm in spite of altered zein composition. W22 contains a more vitreous kernel than the HiII transformable genotype does, in part caused by a duplicated 27 kDa gamma‐zein, a known Mo2 vital to QPM breeding. F1 plants were self‐pollinated and the resulting F2 progeny were screened for both edited alleles as well as the transgene. Plants which were determined to be transgene‐free were carried forward for further analysis. In order to identify lines which contain homozygous edited alleles and a reduced 19 kDa‐zein phenotype, SDS‐PAGE was performed on eight random kernels from several F2 ears. Two of the lines, named 19z‐10 and 19z‐11, were found to have a consistent SDS‐PAGE phenotype across kernels, indicating a homozygous edited allele and the associated reduction in 19 kDa zeins (Figure 3). Importantly, these two lines had differing PCR marker band sizes, indicating distinct editing events (Figure 2).

### Multi‐target editing of the 19 kDa alpha‐zein family generated large chromosomal deletions

In order to precisely characterize the mutation at the edited loci, whole genome single molecule sequencing (PacBio) was used. Due to the repetitive nature of tandem duplicate loci and the high number of double‐strand break sites at the edited loci, long‐read sequencing was determined as the best strategy to accurately characterize the alleles. A local‐assembly approach was used to characterize edited alleles. SMRT reads were aligned to the B73v5 reference genome using three different aligners: NGMLR, lra, and minimap2. Any reads which had alignments in the regions of respective zein loci were extracted from the original fastq and assembled with canu to form a contig covering the edited region. After aligning the contig with lra, structural variants were called using sniffles and pbsv, followed by manual observation in IGV to confirm breakpoints.

**Figure 4:**
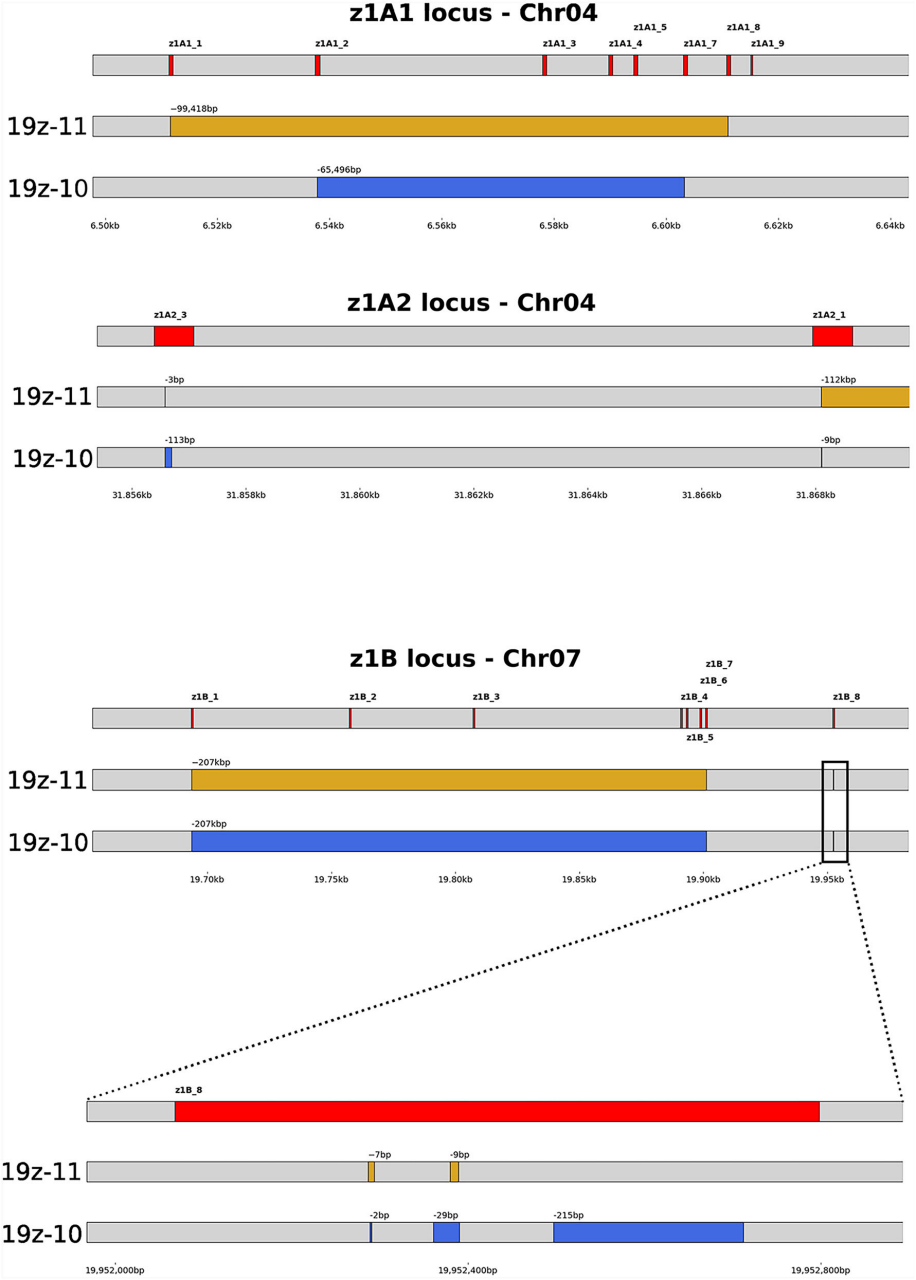
Long‐read sequencing showed large deletions of the 19 kDa alpha‐zeins. The blue and orange highlights denote the region deleted from the respective lines. Each deletion is annotated with the number of base pairs.

The results demonstrated large deletions spanning the alpha‐zein loci (Figure 4). In the A1‐family on chromosome 4, 19z‐10 displayed a >65kb deletion that included all but two family members, and edited alleles of the A2‐family members. Edited line 19z‐11 had similar results, including a >99kb deletion spanning the entire A1‐family, and edits in all A2‐family members. Both lines contained a large deletion in chromosome 7's B‐family, spanning about 207kb, however, each line had unique edits in the remaining z1B_8 gene copy. As expected, no editing was detected in the 22 kDa family members, located upstream of the A1‐family on chromosome 4.

### 19z‐10 and 19z‐11 had reduced 19 kDa alpha‐zein expression and global changes to the endosperm Tran‐scriptome

The transcriptional changes in the edited lines were compared using both wild‐type inbreds in their pedigree, W22 and HiII. RNA was extracted from developing kernels, 20 days after self‐pollination. Quantified RNAseq reads indicated minimal expression of 19 kDa family members in both 19z‐10 and 19z‐11. Normal expression was observed in the 22 kDa family members which together with edit characterization data, provides confidence that the closely related gene family was unaffected despite sequence similarity with the targeted genes.

While genes were differentially expressed between the edited and wild‐type lines, the composition of the DEGs was notably different from that seen between *o2* maize and wild type. Based on an experiment by Zhan et al 2018 (Zhan *et al*., 2018), over 1800 were differentially expressed between wild‐type maize and maize with the *o2* allele, including 186 direct targets identified with ChIPseq. Of the direct O2 targets, only two non‐zein genes were found to be downregulated in the edited lines. Additionally, three direct O2 targets, downregulated in *o2*, were upregulated in the edited lines. This highlights the ‘non‐*o2* based’ feature of 19z‐10 and 19z‐11. Of those >1800 indirect O2 targets (ie. genes differentially expressed in *o2* but not implicated in ChIPseq), 31 non‐zein genes were differentially expressed in both the edited lines and *o2* (Table 1). Additionally, 13 of those 31 were differentially expressed in the same direction in both sets.

**Table 1:** Differentially expressed genes identified in both o2 (Zhan *et al*., 2018) and the 19 kDa edited maize.

Gene	logFC	Zhan 2018 logFC	annotation
Zm00001eb267570	−4.25	−7.78	
Zm00001eb034220	2.75	−5.34	
Zm00001eb026530	5.64	−3.42	
Zm00001eb022980	2.51	−3.21	Proline dehydrogenase domain
Zm00001eb026460	7.58	−3.2	
Zm00001eb163240	2.52	−2.89	Glutamine amidotransferase type 2 domain
Zm00001eb305560	−3.80	−2.86	Heavy metal‐associated domain
Zm00001eb305450	−5.94	−2.52	Mitochondrial substrate/solute carrier
Zm00001eb376570	3.36	−2.46	GDSL lipase/esterase
Zm00001eb098660	2.10	−2.07	
Zm00001eb106160	5.06	−1.92	Sugar phosphate transporter domain
Zm00001eb293820	3.38	−1.77	Serine–threonine/tyrosine‐protein kinase
Zm00001eb388260	1.79	−1.73	Phosphoribosyltransferase C‐terminal
Zm00001eb031090	8.23	−1.67	
Zm00001eb431500	−3.07	−1.56	NAD‐dependent epimerase/dehydratase
Zm00001eb117350	−5.84	−1.55	Zinc finger
Zm00001eb330850	2.68	−1.54	NAC domain
Zm00001eb179200	3.00	−1.32	
Zm00001eb039590	1.93	−1.21	Signal transduction histidine kinase
Zm00001eb291480	−4.14	−0.95	Cystatin domain
Zm00001eb117100	1.93	−0.89	Transketolase‐like
Zm00001eb323590	2.48	0.91	Protein of unknown function DUF538
Zm00001eb032970	−1.98	1.13	Alpha/beta hydrolase
Zm00001eb007320	3.41	1.16	
Zm00001eb146130	1.71	1.29	UspA
Zm00001eb377400	−4.46	1.42	Early nodulin 93 ENOD93 protein
Zm00001eb303510	−2.91	1.51	seed storage helical domain
Zm00001eb421710	5.69	1.52	
Zm00001eb068310	3.74	1.67	
Zm00001eb048140	3.35	1.92	Thaumatin family
Zm00001eb399220	2.28	2.36	Pyridoxal phosphate‐dependent decarboxylase

RNAseq allowed investigation of the transcriptomic changes that occur during proteome rebalancing. Comparison of all reps from 19z to 10 and 19z to 11 were compared to those from HiII and W22, resulting in 1,150 differentially expressed genes (DEG) at an FDR less than 0.05. In order to account for potential false positives caused by differences in pedigree, DEG from a comparison of HiII and W22 were removed, resulting in 661 DEG between the edited lines and wild type (Table S2). GO term enrichment analysis was performed separately on the DEG upregulated in edited lines and those upregulated in wild type. In the edited line upregulated DEG, 37 GO terms were enriched. In wild‐type upregulated DEG, a single GO term, nutrient reservoir activity, was enriched; this is expected given the inclusion of the alpha‐zein storage proteins in ‘nutrient reservoir activity’.

### Segregating combinations of edited loci displayed variability in kernel phenotype

**Figure 5:**
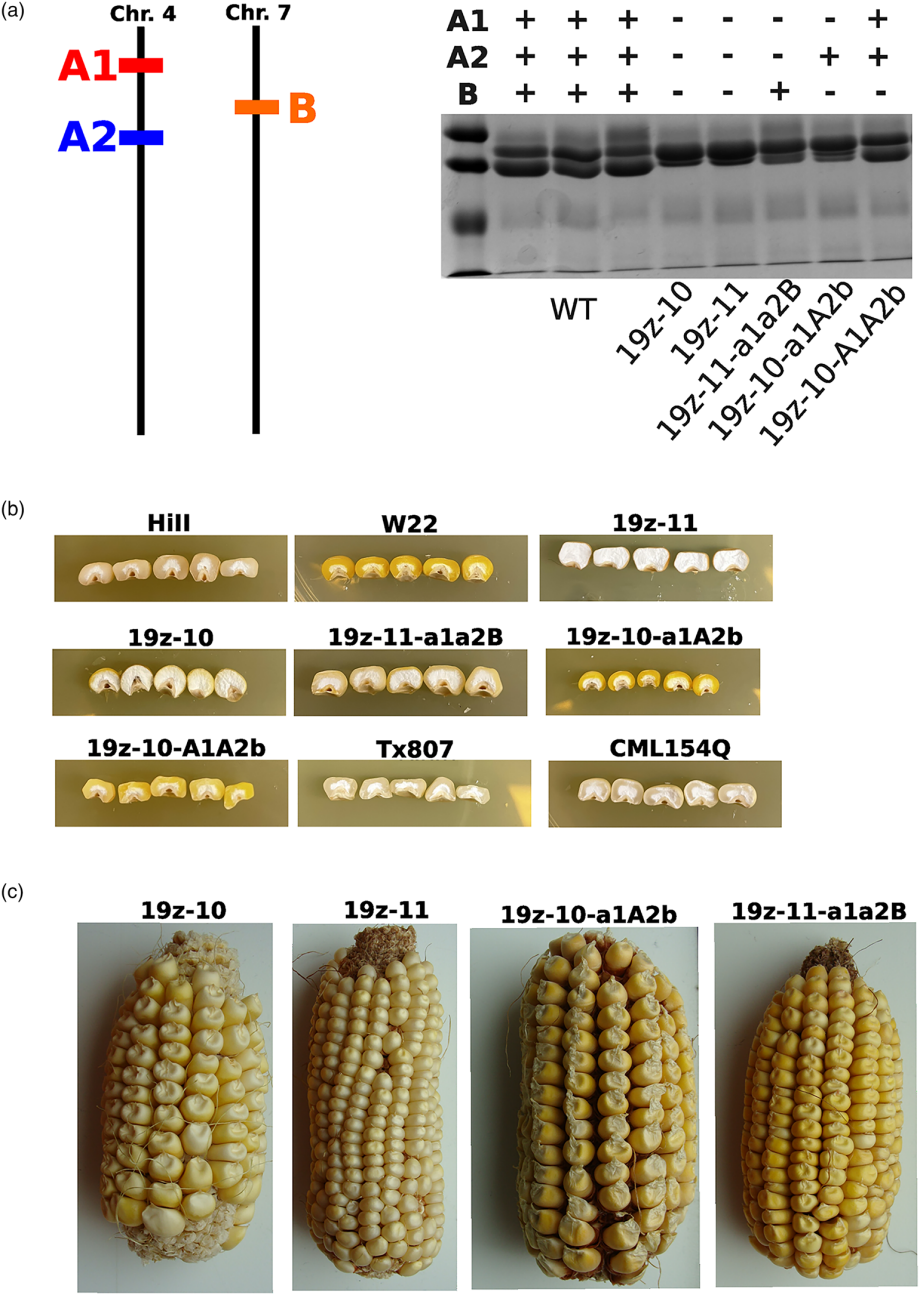
(a) the chromosomal locations of the editing targets allowed for the discovery of segregating lines. (b) Kernel phenotypes of edited lines, wild type and QPM. Partially edited lines had comparable vitreousness to wild type.(c) images of whole ears from F3 lines. Images are not to scale.

Due to the chromosomal locations of the A1, A2 and B alpha‐zein loci, segregant lines of various combinations of edited and wild‐type alleles were available (Figure 5a). During SDS‐PAGE screening of F2:F3 individuals, ears were found that displayed consistent zein phenotypes which were different from the ears determined to contain edited alleles at all three alpha‐zein loci. PCR screening of these ear's progeny revealed these lines were homozygous for the edited allele at some, but not all, alpha‐zein loci. This allowed investigation of the phenotypic effects caused by partial editing of the 19 kDa alpha‐zeins.

The two lines containing edited alleles in all three loci, 19z‐10 and 19z‐11, tended towards an opaque, floury endosperm relative to the wild type (Figure 5b). However, some lines containing a partial array of edited loci displayed a kernel phenotype similar to the wild type, presumably due to having higher levels of residual zeins. In addition to variability in kernel texture, segregants displayed variability in the presentation of zeins on SDS‐PAGE (Figure 5a). While SDS‐PAGE does not provide quantification of protein levels, observing the differences in band abundance provides insight into the contribution of each locus to the overall composition of the 19 kDa alpha‐zeins. The lines containing edited alleles at all three loci, 19z‐10 and 19z‐11, displayed more prominent 22 kDa bands than those of wild type, indicating a rebalancing of the 22 kDa zein in the absence of the 19 kDa zeins. The same phenomena were observed when targeting the 19 kDa family with RNAi (Huang *et al*., 2004). However, the amount of 22 kDa rebalancing was less in lines containing partially edited 19 kDa.

**Figure 6:**
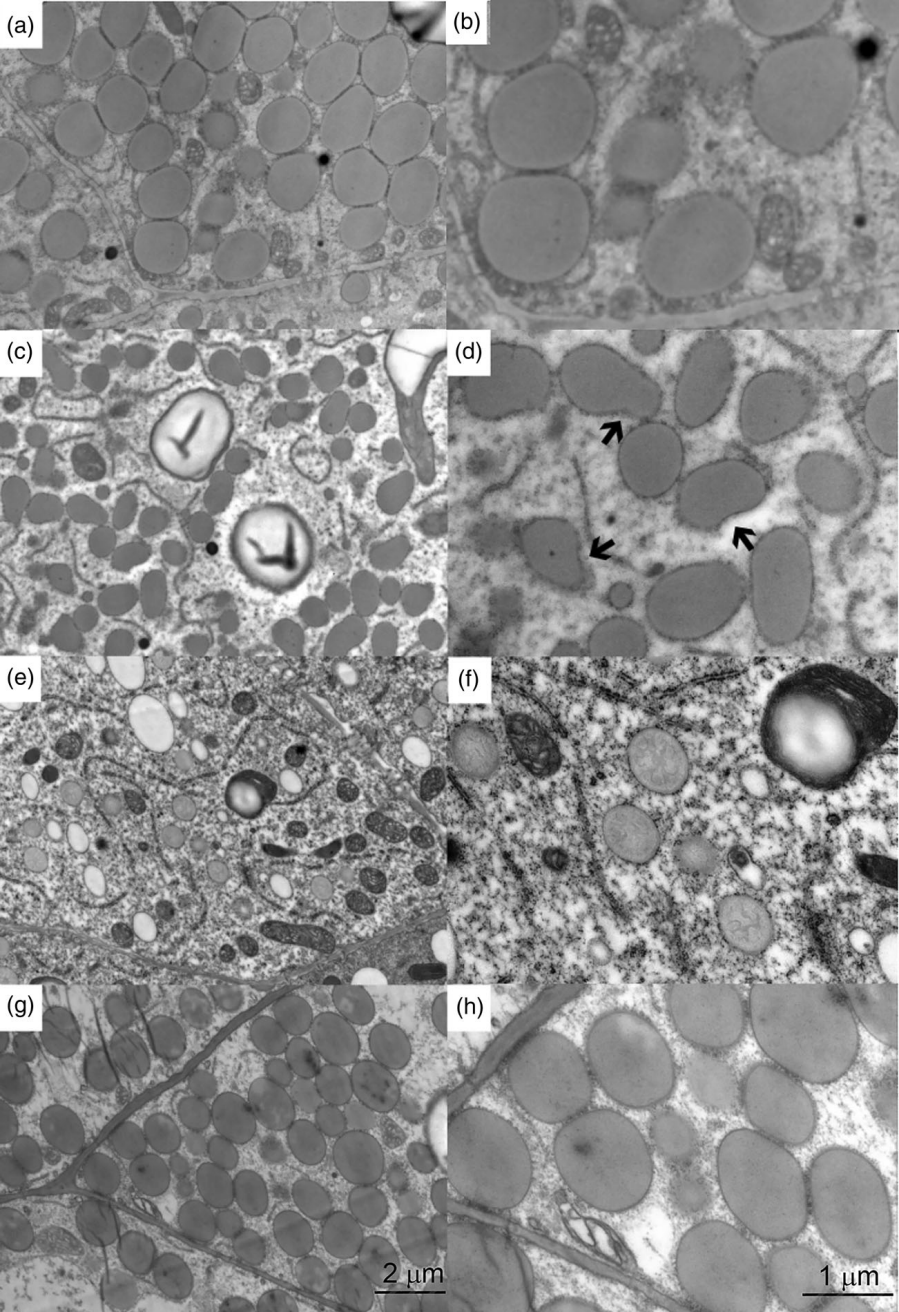
TEM images of 20DAP endosperm. a and b show wild type W22. c and d show a total 19 kDa knockout. 19z‐10. Arrows denote malformed protein bodies. e and f show partial edit 19z‐10‐a1A2b, which has a wild family. g and h show 19z‐11‐a1a2B, which has a wild type B family. a, c, e, and g are at 10000x magnification. b, d, f, and hare at 25000x magnification.

To investigate the changes in protein body morphology brought on by editing the 19 kDa alpha‐zein, 20DAP kernels were viewed with TEM (Figure 6). Protein bodies in 19z‐10 displayed reduced size relative to wild‐type W22 (Figure 6a,b). Additionally, 19z‐10 protein bodies had a malformed, lobed appearance. Protein bodies with this appearance have also been observed in the endosperm of maize containing an RNAi construct targeting the 22 kDa alpha zein, but with functional 19 kDa. The lobed appearance is in contrast to the protein bodies in 19z‐10‐a1A2b and 19z‐11‐a1a2B, whose protein bodies had a reduced size but a normal spherical appearance (Figure 6c,d). However, 19z‐10‐a1A2b displayed a much lower density of protein bodies than the other imaged lines. The protein bodies in this line contain what is possibly residual A2 family 19 kDa zein. 19z‐11‐a1a2B, which contains edited A1 and A2 families but a functional B family, had normally shaped protein bodies that showed a variability in size; some of the protein bodies were the size of wild‐type while others were reduced.

Immunogold labelling of the 22 kDa and 19 kDa alpha‐zeins respectively, as well as other zeins such as the gamma‐ and beta‐zeins, has demonstrated that the various zein families have different protein body localization and thus differing functions in the formation process. For example, the 22 kDa alpha‐zein is localized in an outer ring that surrounds the 19 kDa alpha‐zein core. Additionally, the 27 kDa gamma‐zein contributes to the initial stages of protein body formation, and in mature protein bodies, the outermost surface is primarily made up of the 27 kDa zein. The variability in both kernel texture and protein body appearance between the 19 kDa segregants indicates the possibility that the three 19 kDa alpha‐zein families have evolved different functions in protein body formation.

### Partial knockout of 19 kDa alpha‐zeins led to a greater increase in lysine than total knockout

To quantify amino acid changes in the edited lines, mature kernels from multiple F3:F4 ears were ground into flour. As a control, individuals of the two normal maize lines that were used as parents, W22 and HiII, were measured. Additionally, two QPM inbred lines were measured, Tx807 and CML154Q, in order to compare the non‐*o2‐*based edited lines to high lysine maize varieties possessing the *o2* mutation.

**Figure 7:**
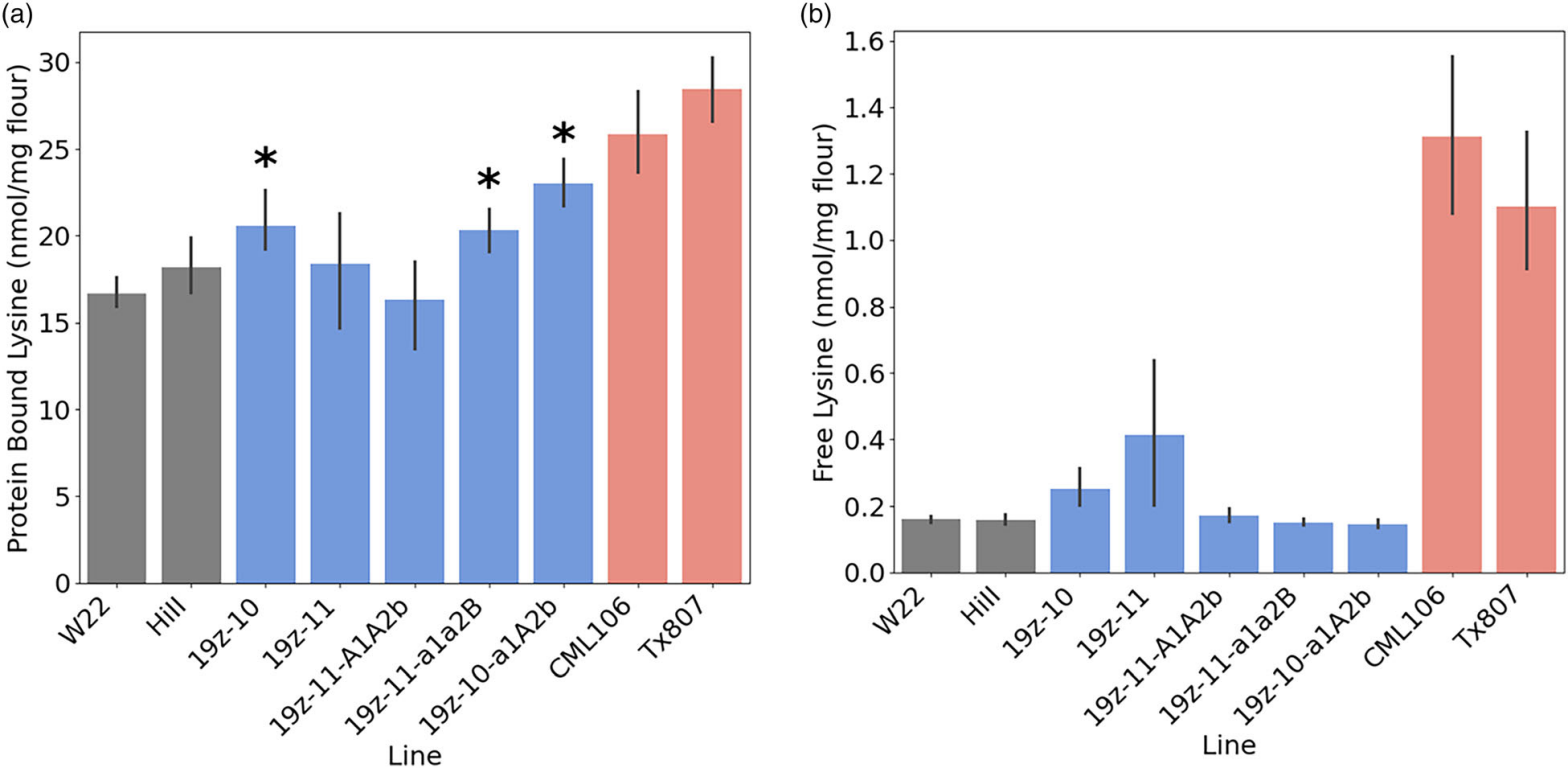
Lysine levels in flour from normal maize, edited, and QPM. *p <0.05.

In comparison to normal, wild‐type maize, three of the five tested lines displayed a significant increase in protein‐bound lysine (Figure 7b). Interestingly, the largest increase was observed in a partially edited line, 19z‐10‐A1a2b, with 32% more protein‐bound lysine than wild type (Table 2). Additionally, 100 kernel weight was unchanged between 19z and 10‐A1a2b, QPM and W22 (Table 3). HiII demonstrated a higher 100 kernel weight than the other lines, likely due to hybrid vigour (all other lines tested are inbred). By comparison, QPM displayed a 54.9% increase in protein‐bound lysine compared to wild type. Free lysine was unchanged in the edited lines, whereas QPM displays a several‐fold increase in free lysine (Figure 7b). This is due to *Opaque2*'s role in lysine degradation. The O2 transcription factor regulates lysine ketoglutarate reductase (LKR), which catabolizes free lysine (Kemper *et al*., 1999), hence the *o2* mutant QPM has reduced LKR and an increase in free lysine. The non‐*o2‐*based edited lines possess increased protein‐bound lysine as a result of proteome rebalancing in response to reduced levels of alpha‐zeins, but the free lysine is unchanged, highlighting the pleiotropic effects of *o2*. Of note, free lysine makes up a small fraction of the total lysine, relative to protein‐bound lysine, in either QPM or wild‐type maize.

**Table 2:** Differences in protein‐bound lysine compared to wild type.

Line	Protein‐bound lysine	*P*‐value
19z‐10	20.5840	0.004
19z‐11	18.3600	0.570
19z‐11‐A1A2b	16.3140	0.401
19z‐11‐a1a2B	20.3220	0.004
19z‐10‐a1A2b	23.0270	2.842e‐05

**Table 3:** Differences in 100 kernel weight.

Line	100 kernel weight (g)	*P*‐value
19z‐10‐a1A2b	21.80	–
Tx807	21.45	0.656
CML106	19.84	0.459
W22	21.90	0.956
HiII	29.60	0.043

**Figure 8:**
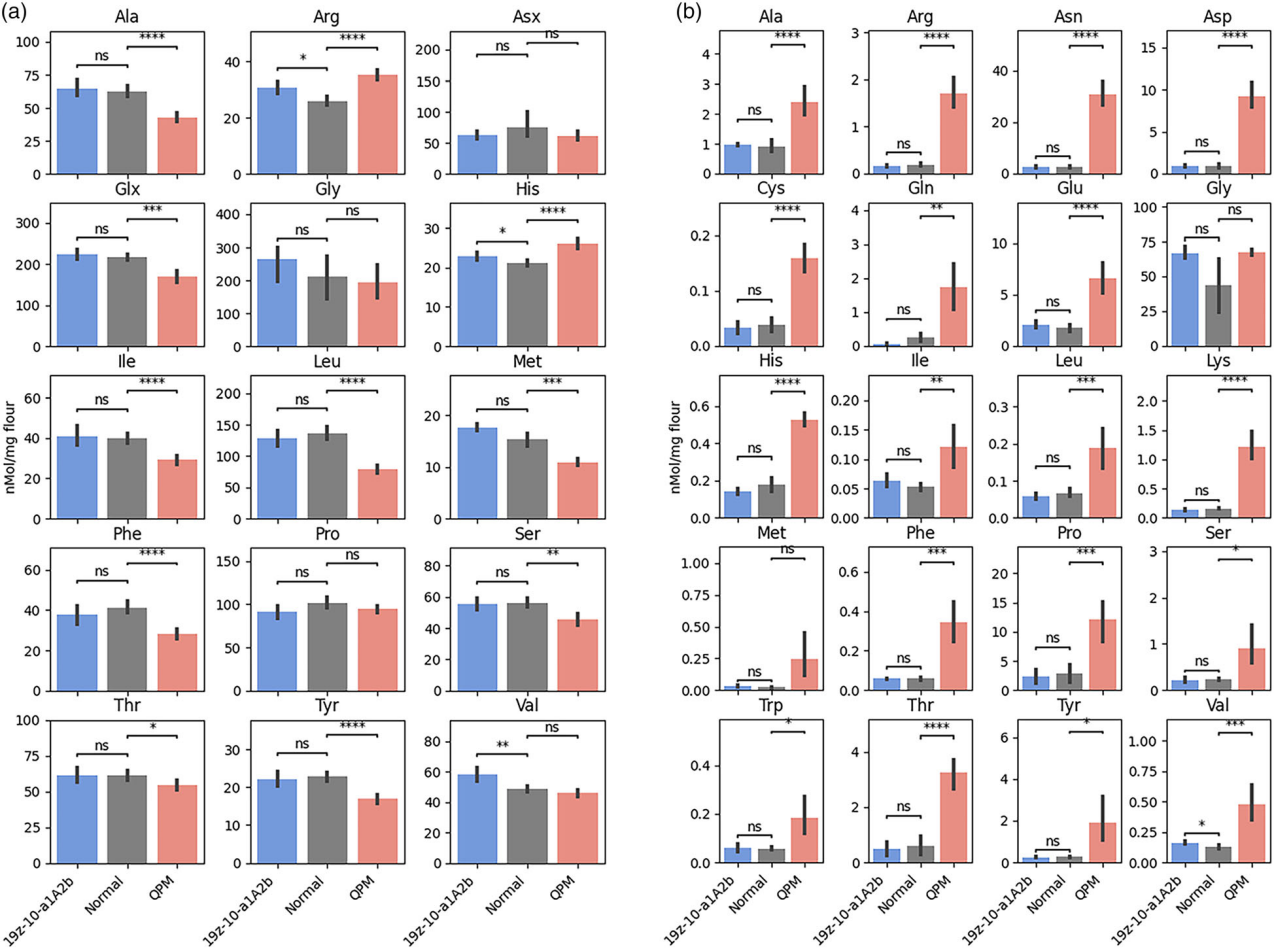
Amino acid levels in flour from normal, edited, and QPM. (a) Protein‐bound amino acids (b) free amino acids.

Despite a lesser increase in lysine relative to QPM, 19z‐10‐a1A2b displayed a more balanced amino acid profile. Of the 15 protein‐bound amino acids tested, other than lysine, nine which were decreased in QPM were unchanged in 19z‐10‐a1A2b (Figure 8a). Valine, an essential amino acid, was increased in 19z‐10‐a1A2b but unchanged in QPM. Another essential amino acid, Methionine, was unchanged in 19z‐10‐a1A2b but reduced by 28.5% in QPM. Other essential amino acids that were higher in 19z‐10‐a1A2b relative to QPM were leucine, isoleucine, threonine and phenylalanine. The collateral reduction of other amino acid levels in QPM germplasm has been observed previously and is attributed to effects of the *o2* mutation (Scott *et al*., 2004). Alpha‐zeins are rich in leucine, isoleucine and phenylalanine, therefore a mutation in zein regulating *o2* leads to a reduction in those amino acids. Other zein proteins, such as the 15 kDa beta‐zein, are rich in methionine. Given the 15 kDa beta‐zein is also regulated by O2, an *o2* mutant could be expected to have reduced methionine. Despite the decreases observed in QPM's protein‐bound amino acids, increases were observed in 19 of the 20 free amino acids measured (Figure 8b). This is in contrast to 19z‐10‐a1A2b, in which the levels of all 20 free amino acids remained unchanged relative to wild type. Changes in QPM‐free amino acid content are related to O2, which regulates genes such as *aspartate kinase 2* (*Ask2*) (Wang *et al*., 2001, 2007). The contrasting response in free amino acid content between QPM and the edited lines furthers the hypothesis that the *o2* mutation is responsible for the free amino acid changes observed in QPM, while the protein rebalancing results from the absence of zeins. These results indicate that selective knockout of an alpha‐zein family confers an increase in lysine without the collateral changes that occur in the presence of a mutant *o2* allele.

## Discussion

### Novel‐edited alleles achieve enhanced lysine and selection‐free recovery of kernel hardness

Achieving enhanced lysine maize with recovered kernel hardness was first accomplished by CIMMYT researchers by several generations of recurrent selection. The results presented here demonstrate that editing the 19 kDa alpha zein can produce enhanced lysine maize, with comparable kernel hardness, with minimal selection. Perhaps as expected, vitreousness was greater in segregating lines containing wild‐type alleles at some of the target loci. Unexpectedly, a segregant line containing an edited allele in two of the three target loci contained the highest increase in lysine. It was observed with SDS‐PAGE that the total 19 kDa alpha‐zein knockouts had rebalancing in the 22 kDa alpha‐zein, whereas less rebalancing was observed in partially edited segregants. This is seen in SDS‐PAGE as a residual 22 kDa band in the non‐total knockout lines. It is possible that the rebound in the 22 kDa is preventing rebalancing towards lysine‐containing proteins, hence the higher lysine observed in segregating lines relative to complete edits. Additionally, editing the zeins directly allows lysine enhancement with a functional *o2* transcription factor.

The most promising line is 19z‐10‐a1A2b, due to its enhanced lysine and vitreousness comparable to QPM. This line contains knock‐out of the A1 and the B family, but retains a functional A2 locus. SDS‐PAGE implies little to no rebalancing of the 22 kDa alpha zeins, perhaps contributing to the lysine enhancement relative to 19z‐10 and 19z‐11. Additionally, TEM of developing kernels demonstrated the protein bodies in this line, while reduced in size, retain a circular conformation relative to 19z‐10. This difference in protein body morphology may be contributing to the retention of vitreousness in 19z‐10‐a1A2b relative to the floury total knockouts.

### Editing the 19 kDa alpha‐zein caused fewer collateral changes to amino acid levels than *opaque2‐*based QPM

The total 19 kDa knockout edited lines 19z‐10 and 19z‐11 exhibited very little similarity in their transcriptional profile than *o2* maize when compared to wild type. Additionally, while edited line 19z‐10‐a1A2b had just 85% of the lysine as QPM, however, the line did not suffer the collateral decreases to non‐lysine amino acids as *o2* mutant QPM. *o2* regulates many pathways in the maize kernel. The lack of zein synthesis is the causative pathway leading to enhanced lysine, through rebalancing of non‐zein proteins. Directly editing alpha‐zeins allows this mode of amino acid changes to take place, without the pleiotropic changes associated with *o2*. However, free amino acids were increased in QPM, a known phenomenon believed to be caused by O2's regulation of amino acid catabolism pathways. Of interest to livestock diet formulation is the reduction of crude protein while still meeting nutritional demands, owing to environmental and economic benefits of low protein diets (Liu *et al*., 2021; Rocha *et al*., 2022). Maize with enhanced lysine and little change to other amino acid levels holds the potential for altering the ratio of corn to soybean meal in diets, and hence lower levels of crude protein without loss of amino acid balance.

In addition to the unaffected non‐lysine amino acid profile, the edited lines did not display a change in the transcription of most O2 direct targets. O2 regulates several important genes. This includes direct targets known to cause negative pleiotropic effects, such as: *b‐32*, a gene involved in pathogen defence; *PPDK*, involved in carbon metabolism; and lysine ketoglutarase, involved in the catabolism of lysine (Zhan *et al*., 2018). None of those direct targets of O2 were observed to be downregulated. Importantly, neither of the two downregulated direct targets were transcription factors. As O2 regulates a network of transcription factors, an *o2* mutation affects a wide range of gene networks that are indirectly affected. In the edited lines, just 35 of the >1800 indirect targets were differentially expressed, a result of those gene regulatory networks remaining intact.

### The three 19 kDa alpha‐zein loci targeted may have evolved distinct functions in protein body formation

Historically, the 19 kDa alpha‐zeins have been considered a single group due to SDS‐PAGE migration as well as sequence similarity analysis indicating a single duplicative origin. However, the lack of targeted mutants and the implications of sequence similarities to RNAi have made differentiating the functions of the A1, A2, and B subfamilies difficult. Segregants derived from outcrossing displayed differences in kernel texture, amino acid change, and protein body morphology. Potentially, this is due to the evolution of different functions of the 19 kDa subfamilies. It has been observed that protein body localization, and its role in kernel protein body development, differs between the classes of zein proteins^33^. The segregating lines developed here can be used to study the functions of the individual 19 kDa subfamilies.

### Transcriptome changes in the presence of 19 kDa alpha‐zein reduction

RNAseq revealed alterations in the transcriptome in the presence of edited alpha‐zein alleles. Notably, an upregulation of genes involved in oxidative stress response was observed. Indeed, the floury centre of normal maize endosperm is subjected to hypoxic conditions (Rolletschek *et al*., 2005) and genes related to hypoxic stress are upregulated in the floury centre of normal maize (Gayral *et al*., 2017). The upregulation of oxidative stress response genes in 19z‐10 and 19z‐11, both of which possess floury kernels, is consistent with this. Alternatively, many genes in this category, in particular the Hsps, are consistent with the unfolded protein response. The edited lines here had upregulation of several Hsp20 family members, but downregulation of an Hsp70 and Hsp90, when compared to normal maize. It has been observed that QPM, when compared to non‐QPM *o2*, has downregulation of several Hsp's (Guo *et al*., 2012). The proposed reason behind this difference between QPM and *o2* is a modifying allele possessed by QPM that increases the expression of a cytosolic PFPα, thus resulting in more efficient ATP production in anaerobic conditions (Li *et al*., 2020). An improved energy balance in QPM was postulated as one of the predominant mechanisms of kernel vitreousness restoration. The availability of ATP facilitates proper protein folding in the ER, and the formation of protein bodies. Of interest is the observation that a partial reduction in 19 kDa aided in restoring vitreous endosperm. Based on protein body formation, it is possible that a partial 19 kDa reduction resulted in a level of hypoxic stress similar to wild type, hence there was no need for modifying alleles. Alternatively, a partial reduction in 19 kDa may result in less ER stress and thus less demand for ATP‐consuming unfolded protein response, relative to the total 19 kDa knockout in 19z‐10 and 19z‐11. Of note, five of the six Hsp20 genes upregulated in edited lines also belong to the enriched GO term ’endoplasmic reticulum lumen’. In addition to the Hsp20, two protein disulfide isomerases, *PDI1* and *PDI2*, were upregulated. *PDI1* has been shown to be upregulated in floury maize mutant *fl2*, which has abnormal 22 kDa alpha‐zeins (Li and Larkins, 1996). It has been proposed that *PDI1* functions as a chaperone during protein body assembly.

Eight genes contributed to the enrichment of GO terms ‘structural constituent of cell wall’ and ‘plant cell wall’ in edited line upregulated DEG. A possible cause is altered cellular differentiation during development between normal and edited maize. Two genes involved in kernel development, *ESR6* and *BETL3* were downregulated in the edited lines (Dai *et al*., 2021). *ESR6*, or embryo surrounding region 6, is involved in the formation of the interface between the embryo and endosperm and nutrient transfer across the two. *BETL3*, or basal endosperm transfer layer 3, is involved in the differentiation of endosperm tissues during development.

Despite no incidental editing occurring, the 15 kDa zein transcript was downregulated in the edited lines. It has been previously observed that RNAi targeting the 22 kDa alpha‐zeins results in reduced 15 kDa transcription, albeit with no reduction in 15 kDa protein levels (Guo *et al*., 2013). Despite an observation of increased 22 kDa alpha‐zein on SDS‐PAGE, transcription did not appear affected. However, the lysine change associated with 19z‐10 and 19z‐11 relative to partial editing is consistent with intra‐zein rebalancing.

## Conclusion

Partial editing of the 19 kDa alpha‐zein resulted in maize kernels with enhanced lysine and vitreous endosperm. Surprisingly, total knockout of the 19 kDa resulted in little to no increase in lysine, perhaps explained by the apparent rebalancing of the 22 kDa alpha‐zeins. The editing strategy employed here is a proof of concept that may allow the conversion of elite‐yielding hybrids to quality protein maize while retaining a hard kernel. Additionally, a functional *o2* transcription may alleviate deleterious consequences brought by the pleiotropic effects of *o2* mutants. For example, *o2*‐free edited lines displayed wild‐type levels of non‐lysine amino acids, whereas *o2*‐containing QPM had collateral changes in the overall amino acid profile. Field trialling can test whether these lines perform better than *o2*‐containing QPM in metrics such as yield and disease resistance. Additionally, the segregants produced here hold value to further study of maize protein body formation, endosperm development, and proteome rebalancing.

## Methods

### Target site selection

A custom Python script was used to extract all sequences with a 5′‐NGG‐3′ PAM sequence and make pairwise comparisons between them. A transcriptomic study was conducted to prioritize gene copies by editing potential importance (Hurst *et al*., 2021). Seed sequence match, location of mismatches and off‐target potential were considered. A set of 6 gRNA sequences was selected which could edit the target sites of all high‐priority gene copies.

### Assembly of binary vector pPTN1585

The plant selectable marker and the Streptococcus pyogenes Cas9 endonuclease expression cassettes, along with the multiplex guide element were synthesized (GenScript Inc, Piscataway, NJ, USA), with the Cas9 ORF codon optimized for sorghum, and each element domesticated for GoldenBraid assembly (Sarrion‐Perdigones *et al*., 2013). The npt II selectable marker cassette is regulated by the 35S CaMV promoter while the Cas9 expression is governed by the maize ubiquitin1 promoter coupled with its first intron. The six designed guides are transcribed by the rice OsU6a, OsU6b, and OsU6c, along with the maize ZmU6 and wheat TaU6 small RNA promoters.

The plant selectable marker cassette, along with editing reagents, were dropped into the destination vector pDGB3‐alpha‐Ωvia GoldenBraid assembly (Sarrion‐Perdigones *et al*., 2013) and the resultant binary vector designated pPTN1585.

### Maize transformation

The binary vector pPTN1585 was mobilized into the disarmed Agrobacterium tumefaciens strain C58C1/pMP90 (Koncz and Schell, 1986) via tri‐parental mating, and the derived Agrobacterium transconjugants confirmed via plasmid rescue. Maize transformations were conducted using the maize HiII genotype (Armstrong, 1991) as previously described (Sattarzadeh *et al*., 2010).

### PacBio sequencing

Leaf tissue was sampled from V8‐stage plants. High molecular weight gDNA was extracted using the Nanobind Plant Nuclei Big DNA Kit (Circulomics). All DNA samples passed quantity and quality controls performed using Fluorometer (Qubit, Invitrogen), automated electrophoresis (TapeStation, Agilent) and spectrophotometer (NanoQuant, TECAN), before proceeding to library preparation. HiFi libraries were generated for each sample using the SMRTbell Express Template Prep Kit 2.0 (PacBio). Each library was sequenced in two SMRTcell‐8M on the Sequel‐IIe in HiFi‐CCS mode. Reads were aligned with lra. Reads that aligned in the region of the alpha‐zeins +/− 1MB were extracted and assembled with canu. The assembled contigs were aligned again with lra and variants were called using Sniffles and viewed on IGV.RNAseq.

Fifteen developing kernels were sampled 20 days after pollination for each sample. Three biological replicates were used for each of four genotypes: the two edited lines and the two parent genotypes (HiII and W22). The kernels were ground and RNA was extracted using Trizol method. All 18 RNA samples were analyzed on the Bioanalyzer (Agilent) and quantitated using Qubit (Invitrogen). All extracts showed an RNA Integrity Number (RIN) > 6, suitable for RNA‐Seq analysis on the Illumina workflow. RNA‐Seq libraries were constructed using the TruSeq Stranded mRNA kit (Illumina) following the manufacturer's instructions. Completed libraries were quantitated using qPCR (BioRad®) and their sizes were visualized on the Bioanalyzer (Agilent®). All libraries passed QC with no traces of contaminating adapter dimers and a range of sizes spanning 250–700 bp with an average peak of about 280 bp. The sequencing was performed using NovaSeq 6000 to generate 150 bp paired‐end reads. Reads were aligned with HISAT2 (Kim *et al*., 2019) and quantified with stringtie.

### Sanger sequencing of A1/A2 bands

PCR using the A‐family primers was performed on a HiII control, 19z‐10, and 19z‐10‐a1A2b. Gel‐purified reactions were inserted into the pCR2.1 vector and colony PCR obtained samples of each product produced by each genotype. Capillary sequencing reads were aligned to individual A family alpha‐zein gene sequences as well as the contigs obtained from PacBio sequencing to determine the haplotype present in each line.

### Amino acid quantification

For amino acid analysis, four biological reps (ie. individual plants) were sampled for each maize line. Two separate pools of 30 mature kernels from each biological rep were ground into flour using a Tissue lyzer. The amino acid value for each biological rep was considered the average of the two separate 30 kernel samples. Free and protein‐bound amino acids were extracted and analyzed separately, according to Angelovici *et al*. (2013).

## Conflict of interest statement

The authors declare no conflict of interest.

## Author contributions

JPH and DRH wrote the manuscript. JPH carried out breeding and introgression activities and performed bioinformatics analysis. TF assisted in lab analysis. SS and TC performed all transformation activities. YZ performed microscopy work. AY and RA performed amino acid analysis.

## Funding information

This work is supported by the AFRI Foundational Program [Grant no. 2021‐67013‐33831/project accession no. 1025334] from the USDA National Institute of Food and Agriculture.

## Supporting information


**Table S1** ‘Sequences of the six gRNA used in this study’.

**Table S2** ‘Differential expression results of wild‐type and edited 20DAP kernels’.

## Data Availability

Sequencing data from the PacBio WGS as well as RNAseq has been deposited in [PRJNA1041363].
